# Social and genetic connectivity despite ecological variation in a killer whale network

**DOI:** 10.1098/rspb.2024.0524

**Published:** 2024-04-17

**Authors:** Eve Jourdain, Richard Karoliussen, Sarah L. Fordyce Martin, Øystein Langangen, Todd Robeck, Katrine Borgå, Anders Ruus, Andrew D. Foote

**Affiliations:** ^1^ Department of Biosciences, University of Oslo, 0316 Oslo, Norway; ^2^ Norwegian Orca Survey, 8480 Andenes, Norway; ^3^ Department of Natural History, NTNU University Museum, Norwegian University of Science and Technologies (NTNU), 7491 Trondheim, Norway; ^4^ Zoological Operations, SeaWorld Parks and Entertainment, Orlando, FL 32819, USA; ^5^ Norwegian Institute of Water Research, 32821 Oslo, Norway

**Keywords:** cetacean, sociality, genetic relatedness, mark–recapture, photoidentification, *Orcinus orca*

## Abstract

Philopatric kin-based societies encourage a narrow breadth of conservative behaviours owing to individuals primarily learning from close kin, promoting behavioural homogeneity. However, weaker social ties beyond kin, and across a behaviourally diverse social landscape, could be sufficient to induce variation and a greater ecological niche breadth. We investigated a network of 457 photo-identified killer whales from Norway (548 encounters in 2008–2021) with diet data available (46 mixed-diet individuals feeding on both fish and mammals, and 411 exclusive fish-eaters) to quantify patterns of association within and between diet groups, and to identify underlying correlates. We genotyped a subset of 106 whales to assess patterns of genetic differentiation. Our results suggested kinship as main driver of social bonds within and among cohesive social units, while diet was most likely a consequence reflective of cultural diffusion, rather than a driver. Flexible associations within and between ecologically diverse social units led to a highly connected network, reducing social and genetic differentiation between diet groups. Our study points to a role of social connectivity, in combination with individual behavioural variation, in influencing population ecology in killer whales.

## 1. Introduction

Patterns of social relationships in animal societies reflect encounter rate and association time among individuals [1]. Inter-individual social relationships can influence inheritance through both social learning and gene flow [2], thereby shaping cultural and genetic evolution [3]. Highly philopatric kin-based societies often encourage a narrow breadth of conservative behaviours owing to individuals primarily learning behaviours from close kin. Theoretically, such a scenario could result in highly correlated social, genetic and ecological variation, the formation of ecotypes, and even incipient ecological speciation [4,5]. However, weaker social ties to a larger social landscape may also play a role in shaping individual behaviour and population-level ecology [6,7].

As a social species adopting a wide range of dietary (and associated behavioural) specializations throughout its distribution, the killer whale (*Orcinus orca*) represents a unique model species to investigate the interplay between kinship, social structure and behaviour [8]. In killer whales, matrilineal sociality promotes behavioural transmission among matriline members through social learning and, ultimately, cultural perpetuation of dietary specializations [9–11]. Social segregation caused by cultural barriers, whereby individuals preferentially interact with others adopting similar dietary specialization, may further restrict social contacts and lead to reproductive isolation among ecotypes [5,12–14].

From matrilineally driven specialization may emerge an increasingly patchy social landscape of behaviourally, genetically and socially assorted groups. The reduced potential for social transmission between groups may further limit variation in behaviour [15]. This could explain why dietary specializations are maintained even if becoming maladaptive, as observed in southern resident killer whales persistently foraging on depleted Chinook salmon stocks [16,17]. In contrast, populations made of interconnected, behaviourally diverse groups should promote individual heterogeneity and greater ecological variation, through a richer information landscape at the population level [15]. However, this has to-date been poorly explored [6,7,18].

In the northeastern Atlantic, killer whale seasonal movement and highly specialized feeding techniques reflect population-level resource specialization on large regional fish stocks e.g. spring-spawning and summer-spawning stocks of Atlantic herring (*Clupea harengus*) off Norway and Iceland, respectively [19–21]. However, field observations and dietary markers rather indicated dietary variations within both the Icelandic and Norwegian populations [22–26]. While some groups appear to be conspicuous fish specialists, others adopt a mixed diet that includes both fish and marine mammals [23,26,27]. This is in sharp contrast with the sympatric, highly specialized fish-eating ‘*resident*’ versus mammal-eating ‘*Bigg's*’ (previously known as ‘*transient’*) ecotypes of the northeastern Pacific, for which 50 years of research revealed no overlap in diet [8]. Atlantic and Pacific killer whales offer contrasting systems, ideal to begin to investigate the interplay between sociality, kinship and behavioural/ecological diversity in killer whales, with broad relevance for other animal societies.

Herein, we used a multi-disciplinary dataset consisting of association, ecological (diet) and genetic data to investigate the relationship between these variables in a multi-decadal studied population of Norwegian killer whales. We conducted (1) a network analysis on mark–recapture data: to quantify patterns in, and identify potential correlates of, association at the dyadic (between pairs of individuals) and network levels, and to assess how individuals with different diets were distributed and inter-connected; (2) a complementary genetic analysis: to investigate potential genetic differentiation between the different diet groups, to detect outcomes of social connectivity that may have occurred over long timescales. We discuss our findings in the light of literature on killer whale sociality and highlight the potential interplay between network structure and population ecological niche breadth.

## 2. Material and methods

### (a) Study population

Data on diet and social affiliations of photo-identified killer whales have been collected across all seasons in Norway. Most individuals were encountered at herring wintering grounds, consistent with herring being an important seasonal prey resource [28]. After the herring departed the wintering grounds, a subset of known herring-eating killer whales was annually observed to switch to feeding on other fish prey (e.g. lumpfish, *Cyclopterus lumpus* [22]). A narrow isotopic niche suggested a fish-specialized diet for these whales [23]. Other Norwegian killer whale groups, however, have been known to feed on both mammalian and fish prey [24,27]. Sighting history and isotopic values for those individuals strongly suggest inter-group variations in the proportions of marine mammals (harbour porpoise, *Phocoena phocoena*, and seals, Phocidae) versus fish prey consumed. Specifically, some groups are known to be mainly feeding on mammals all year round, with only a little feeding on fish, while other groups seem to feed on mammals only seasonally or opportunistically, with fish making most of their diet [23].

### (b) Field methods

Killer whale photographs were collected on board boat-based research surveys conducted in all seasons in northern Norway between 2014 and 2021, and by members of the public (citizen science) between 2008 and 2021 across the entire Norwegian coastline (for details, see electronic supplementary material). For both sources, metadata on date, time and location allowed verification that all individuals identified from a series of photographs were from a unique group (i.e. individuals within a few body lengths of each other and displaying coordinated behaviours); photographs for which group membership was uncertain were discarded. Time period and location associated with a killer whale group defined an encounter, each of which received a unique numerical encounter identifier. Individuals were considered associated if they occurred in the same encounter (electronic supplementary material, figure S1*a*). Between 2017 and 2021, photo-identified individuals were biopsied on an opportunistic basis from the research vessel following the protocols described in [23] (electronic supplementary material, figure S1*b*) (permit FOTS-ID: no. 10176 in 2017–2018, report no. 2016/179856; no. 18146 in 2019–2020, report no. 18/257593; and no. 24249 in 2021, report no. 20/151683). Skin was also sampled from one dead stranded individual. Skin samples were stored at −20 or −80°C until DNA extraction.

### (c) Photo-identification and attributes of individuals

For each encounter, killer whales were identified from photographs using the shape and nicks of the dorsal fin, and scars and pigmentation of the adjacent grey saddle patch (as per Bigg [29]) (electronic supplementary material, figure S1*b*). Photographs were scored for quality, and individuals' sex (*female*, *male*, *unknown*) was determined based on observational evidence of physical and sexual maturity following the methods described in [28]. Photo-identified killer whales were assigned a dietary group based on a decade of individuals’ predation records, seasonal occurrence patterns and skin stable isotopic values (for details, see electronic supplementary material; [22–24,28]).

### (d) Genetic analysis

Genomic DNA was extracted from skin biopsies collected from wild killer whales using a DNeasy Blood and Tissue kit (Qiagen, Valenica, CA) and from whole-blood samples of three killer whales in managed care using a QIAamp DNA Mini blood kit and following the manufacturer's instructions (Qiagen, Valenica, CA). Genomic DNA was then sheared to an average size of approximately 500 bp using either a Diagenode Bioruptor Pico or a Covaris ME220 sonication device. The sheared DNA extracts were converted to Illumina sequencing libraries using New England Biolabs NEBNext Ultra II library kit. Libraries were subsequently dual-indexed using NEBNext dual-indexing primer pairs and amplified for seven cycles, and purified using NEBNext beads. The DNA concentration of the libraries was measured using an Agilent TapeStation (Agilent Technologies). Libraries were then equimolarly pooled and sequenced across a partial lane of an Illumina Novaseq S4 platform to generate approximately 200 Gb of data and thereby obtain complete mitochondrial genome sequences.

The equimolarly pooled libraries were then subjected to one round of custom enrichment capture using genome-wide biotinylated RNA baits (eight reactions) designed to capture 1346 genome-wide single nucleotide polymorphisms (SNPs) manufactured by myBaits Daicel Arbor Biosciences (see [30]), with a hybridization period of 24 h at 65°C. The custom-designed baits are available from myBaits Daicel Arbor Biosciences using the follow codes, design ID: D10110Orca, reference number: 210810-901. The SNPs were those identified as being polymorphic in a RAD-seq dataset [31] that included killer whale populations from the Atlantic, Pacific and Southern Ocean, and which were physically separated by greater than 100 kb on autosomal scaffolds and in non-repeat, high-mappability regions of the killer whale genome Oorc_1.1 (see [32]; https://datadryad.org/stash/dataset/doi:10.5061/dryad.803q8). The pool of post-capture libraries was then sequenced across a partial lane of an Illumina Novaseq S4 platform to generate approximately 50 Gb of data and thereby obtain medium- to high-coverage genotypes at the enriched SNPs for the samples in our dataset.

Demultiplexed reads from the enriched libraries were processed with AdapterRemoval2 [33] to trim residual adapter sequence contamination and to remove adapter dimer sequences as well as low-quality (*Q* < 30) stretches at the read-ends. Filtered reads greater than 50 bp were then mapped using the BWA-MEM algorithm [34] to the reference assembly (Oorc_1.1; [35]), requiring a mapping quality greater than 30. Clonal reads were collapsed using the rmdup function of SAMtools v. 1.13 [36].

Reads from the shotgun sequenced libraries were processed as above and mapped using BWA-MEM to a fasta file for the mitochondrial genome sequence of a Norwegian killer whale with haplotype ENAHN1 (NCBI accession: NC_023889.1; [37]), and filtered as above, and including only sites that were covered by at least three independent sequencing reads. Mitochondrial sequences were then converted from Bam to Fasta format using the -dofasta option in ANGSD [38]. In a previous study of 139 killer whale mitogenome sequences [37], the inclusion of intra- and inter-lab polymerase chain reaction (PCR), library build, and sequencing replicates identified inconsistencies in the assembly of polynucleotide repeat regions—one of between 9 and 14 Cs in a row (positions 1130–1144 in the original alignment), and another region of seven to eight As in a row (positions 5210–5217). Morin *et al.* [37] therefore shortened these to a fixed set of nine Cs and seven As, respectively, to avoid introducing potentially erroneous variation into phylogenetic analysis in that and subsequent follow-up studies (e.g. [39,40]). We followed this same conservative approach in our mapping of mitogenomes and identification of sequence variation in this study.

Pairwise relatedness, to be used in the network analysis, was estimated from the high-coverage SNP data using ngsRelate2 [41]. This method estimates relatedness between pairs of individuals within a potentially inbred population from genotype likelihoods, and provides robust estimates from depth of coverage as low as 4× [41]. The principal measure of pairwise relatedness used in this study is *r_xy_* as per Hedrick & Lacy [42]. To validate our relatedness estimates of wild Norwegian killer whales, we used relatedness estimates of three Icelandic killer whales living in a managed-care facility (Sea World Orlando, Florida, USA) of known pedigree (electronic supplementary material, figure S2) and originating from the same metapopulation as Norwegian killer whales (see [43]). For these animals, blood samples were collected voluntarily from the peripheral periarterial venous rete on the ventral tail fluke using a 19-gauge winged blood collection set or attached to a vacutainer collection system as part of routine health assessments. Immediately after collection, a subsample of the blood (1 ml) was placed in BD Vacutainers (Becton Dickinson, Franklin Lakes, NJ) containing ethylene diamin tetraacetic acid (EDTA). Samples were inverted in the Vacutainer for a minimum of 10 times and then frozen at −80°C until DNA extraction. DNA extraction, sequencing and library preparation were performed as described above.

### (e) Network analysis

#### (i) Measuring associations

We quantified occurrence and strength of associations among killer whales in Norway from photographic mark–recapture data (for data selection criteria, see electronic supplementary material). Individuals were considered associated if they were photo-identified together in the same encounter (‘gambit of the group’ [44,45], electronic supplementary material, figure S1*a*). Sampling periods were calendar days, with identified associations assumed to last for the entire day. We calculated the simple ratio index (SRI) in the R software v. 4.2.0 [46] using the package asnipe [47]. The SRI gives the probability of two whales being photographed in the same encounter out of the total number of times association would have been possible [48] (see electronic supplementary material). The SRI is an appropriate index when using group membership as association criteria, and when calibration data related to detection biases are not available [49]. Because the results were consistent when adopting a threshold of three or five sightings (see Results), we chose the least restrictive cut-off value of three sightings that led to the highest number of individuals included in the analysis.

#### (ii) Correlates of association between pairs of whales

We explored correlates of association at the dyadic level. A Bayesian generalized linear mixed effects model (GLMM) was fitted to the number of times a dyad was associated (binomial response variable). Possible number of associations (restricted to within weeks and locations to account for individuals' movement) was used as number of trials underlying each observation. Individual and dyad random effects accounted for the dyadic nature of our data [50]. The model was first fitted to the full network (Dataset 1: *n* = 457 whales), with sex and diet similarity as potential predictors of association. The model was then fitted to the 73 whales with genetic data available (Dataset 2), allowing for inclusion of biparental genetic relatedness and matrilinear haplotype as additional predictors. We set normal priors with mean 0 and standard deviation 0.5. The model was fitted in the brms package in R via STAN [51] and ran on four independent Monte Carlo Markov chains (MCMCs), with 4500 iterations each. The first 1500 iterations were used as warm-up and the next 3000 for sampling. Trace and density plots for MCMC draws and an $\hat{R}$ value close to 1 for estimated parameters were used to confirm stationarity, chain mixing and model convergence. Posterior predictive check plots (used to compare the distribution of predicted versus observed data) along with the Bayesian *R*^2^ statistic for regression models were used to assess overall model fit and performance. Effect sizes were estimated from the posterior distributions of parameter estimates, and contrasts were evaluated as pairwise comparisons using the emmeans package in R [52].

#### (iii) Correlates of network subdivision

We identified significant communities (social units) nested within the network. We applied Louvain's community detection algorithm to the modularity matrix of the full network (Dataset 1: *n* = 457 whales; electronic supplementary material, figure S1) in the R package igraph [53,54]. The algorithm divides the network into communities of densely associated dyads, whilst assigning individuals that are only loosely associated to different clusters (see electronic supplementary material). To further explore how mixed-diet killer whales were nested within communities, we applied Louvain's algorithm to individual communities.

We tested whether network subdivision correlated with dyadic variables whilst accounting for uncertainty in individuals' community assignment (owing to a low threshold of three sightings for inclusion in the analysis, and a high number of null association indices in our data). Using the predicted number of associations for each dyad for a sample of 3000 draws, generated from the Bayesian model (Dataset 1: *n* = 457), we calculated SRI values for each dyad for each draw. We then applied Louvain's community detection algorithm to the network (SRI) calculated for each draw, leading to a posterior distribution of community assignments for each whale. We calculated within- and between-community average values of dyadic variables (diet, sex and haplotype similarities and genetic relatedness) for each draw, leading to a posterior distribution of differences in mean values within, relative to and between communities. When 95% of the difference values were greater than 0, we considered the difference between mean similarity values within, relative to and between communities significant.

### (f) Number of populations

Mark–recapture data originated from incomplete sampling of true associations and only reflected social relationships for the study period. We complemented our network analysis with genomic data for insights into social processes that may have occurred over longer time spans (Dataset 3: *n* = 106 whales; electronic supplementary material, figure S1). We ran a non-hierarchical individual assignment analysis in NGSadmix [55] to investigate potential genetic structuring aligning with diet groups. Specifically, we tested if *k* = 2 (populations) fitted our data better than *k* = 1. Based on observed allele frequencies from the 1346 polymorphic putatively neutral SNPs, the proportion of individuals' ancestry attributable to each of the *k* = 2 populations was estimated. We performed 10 independent replicate runs for cross-validation. Best supported model was identified based on highest mean log-likelihood.

## 3. Results

### (a) Data summary

Between 2008 and 2021, a total of 548 encounters led to 3312 good quality photo-identifications for 457 killer whales (215 males, 186 females, 56 unknowns) seen in ≥3 sampling periods (electronic supplementary material, table S1 and figure S1). Of these 457 individuals, 46 were identified as adopting a mixed diet (confirmed to be feeding on both mammalian and fish prey) while the remaining 411 individuals were assigned an (exclusive) fish diet. Individuals, including mixed-diet whales, were encountered in all seasons throughout the multiple years of the study period (electronic supplementary material, table S2). Number of killer whales identified per day ranged from 1 to 27 (mean ± s.d.: 4.8 ± 3.6). Individuals were encountered in 3 to 22 encounters (mean ± s.d.: 5.8 ± 2.9) in 1 to 8 years (mean ± s.d.: 3.5 ± 1.3). Between 2017 and 2021, a total of 106 unique killer whales were biopsy-sampled, of which 73 were included in the network analysis on the basis of ≥3 sightings (electronic supplementary material, figure S1 and table S1).

DNA extraction and sequencing from all skin samples resulted in high depth of coverage (mean ± s.d. = 40.76 ± 19.63; electronic supplementary material, figure S3) at known SNPs targeted by enrichment capture. Of the 1346 genome-wide SNPs targeted in the capture enrichment experiment, 349 were found to be polymorphic in this population (see electronic supplementary material, file for genomic coordinates and allele frequencies). Pairwise relatedness generated from three captive individuals of known relatedness validated that our approach had sufficient power to correctly infer relatedness (electronic supplementary material, figure S2). Average relatedness of the 106 killer whales sampled for this study is reported in electronic supplementary material, figure S4*a* and table S3. Mitogenome haplotypes (16 390 bp) were generated for all but two samples. All sequencing data are accessible at the NCBI BioProject PRJNA956724: MULTIWHALE.

### (b) Network analysis

#### (i) Association indices

**Figure 1.**
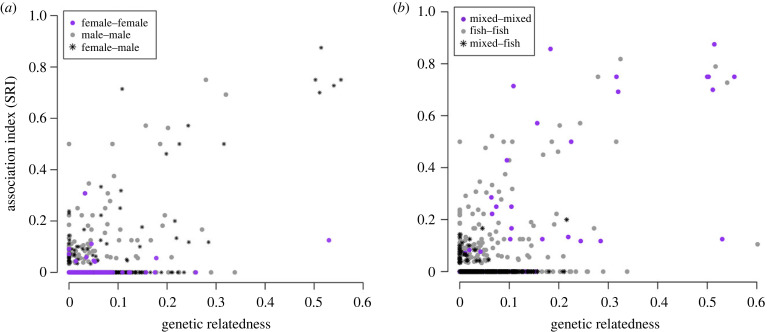
Relationship between social association (simple ratio index, SRI) and biparental genetic relatedness for all pairs of killer whales with genetic data available and retained for network analysis (*n* = 5329 pairs of 73 unique individuals). For each data point (pair of whales), plot shows if individuals are of the same or different sex (*a*) and from a similar or different diet group (*b*). Sample size of non-zero SRI values for sex groups is 86 pairs of males, 10 pairs of females and 58 pairs male–female. Sample size of non-zero SRI values for diet groups is 143 pairs of whales both adopting an (exclusive) fish diet, 26 pairs of whales both adopting a mixed diet (of fish and mammal prey), and 33 pairs of whales adopting different diets (fish versus mixed diet). The zero SRI values correspond to pairs of whales never seen in association in our dataset.

The full network (Dataset 1: *n* = 457 whales) had a non-zero simple ratio index (SRI) of (mean ± s.d.) 0.19 ± 0.19 (range = 0.03–1.00, *n* = 4768 dyads; electronic supplementary material, table S4) and a high percentage (95%) of null values (pairs of whales never seen in association in our dataset). The distribution of non-zero SRIs indicated a high proportion of weak associations compared with fewer strong ties (electronic supplementary material, figure S4*b*). Overall, 501 pairs of whales (10% of the non-zero SRIs) were associated half of the time or more, of which 43 (1%) were seen in constant association (SRI = 1). Strongest bonds were explained by maternal kinship, especially for female–male pairs (figure 1).

#### (ii) Correlates of association between pairs of whales

**Figure 2.**
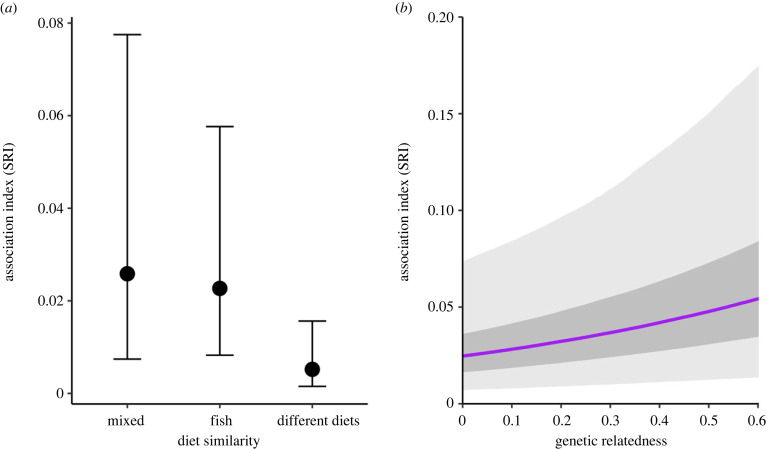
Effect size of (*a*) diet and (*b*) biparental genetic relatedness on structuring pairwise associations as evaluated from the posterior distributions of parameter estimates when fitting the Bayesian model to Dataset 2 (*n* = 73 individuals). Error bars indicate 95% credible intervals (CI) (*a*) and dark- and grey-shaded areas depict 50 and 95% CI, respectively (*b*).

Our first model fitted to Dataset 1 (*n* = 457 whales, *R*^2^_Bayesian_: mean = 0.868, 95% credible interval (CI) = 0.860–0.875; electronic supplementary material, figure S5) revealed that social bond at the dyadic level increased when individuals adopted similar diets (odds ratio = 5.868, 95% CI = 3.984–8.696). A second model fitted to Dataset 2 (*n* = 73 individuals with genetics; *R*^2^_Bayesian_: mean = 0.919, 95% CI = 0.896–0.937; electronic supplementary material, figure S6) showed that social bond increased with both increasing pairwise genetic relatedness (odds ratio = 3.976, 95% CI = 1.526–10.328) and diet similarity (figure 2). Notably, the odds of finding pairs of whales that both adopted a mixed diet (odds ratio = 5.050, 95% CI = 2.224–9.240), or a fish diet (odds ratio = 4.460, 95% CI = 1.676–9.210), were higher than finding pairs adopting different diets (i.e. mixed-diet/fish-diet pairs). In contrast, the odds of encountering fish-diet or mixed-diet pairs did not differ (odds ratio = 1.130, 95% CI = 0.384–2.290) (figure 2*a*). However, associations between pairs of poorly related whales or whales with different diets were also observed (e.g. 33 dyads with different diets showing SRI > 0; electronic supplementary material, figure S4 and table S4). Sex (Dataset 1: odds ratio = 0.938, 95% CI = 0.810–1.084; Dataset 2: odds ratio = 0.658, 95% CI = 0.374–1.160) and haplotype similarity (Dataset 2: odds ratio = 1.502, 95% CI = 0.771–2.936) did not have any effect on dyadic social bond in our data. Results were consistent when applying five sightings as a more conservative threshold for inclusion of individuals in the analysis (electronic supplementary material, figures S7 and S8).

#### (iii) Correlates of network subdivision

**Figure 3.**
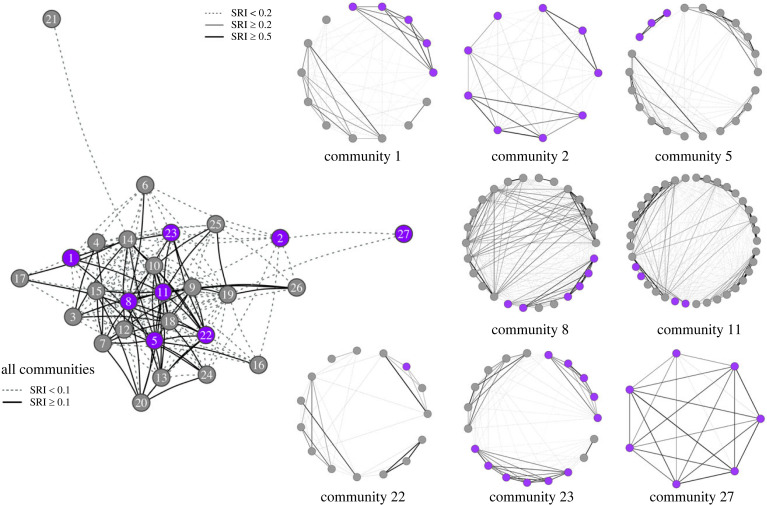
Networks showing how mixed-diet killer whales (*n* = 46) are nested within the larger social network (*n* = 457). The ‘all communities’ network (left) depicts the 27 communities identified with Louvain's community detection algorithm and mean associations between them. Each node (circle) represents one community containing 4 to 37 killer whales, and labels correspond to community IDs as listed in electronic supplementary material, tables S5 and S6. Purple-shaded nodes indicate the eight communities that contain mixed-diet killer whales. Pairwise associations within each of these eight communities are shown as individual sociograms in which nodes represent individuals and with purple-colored nodes indicating mixed-diet killer whales. In all networks, thickness of the edges (ties) relates to the strength of association (simple ratio index, SRI) between pairs of communities/killer whales.

Louvain's clustering technique identified 27 communities for a modularity value of *Q* = 0.68 (Dataset 1: *n* = 457 whales; figure 3 and electronic supplementary material, table S5). Number of individuals assigned to each community varied between 4 and 37 (mean ± s.d.: 27 ± 9). Killer whales known to feed on mammals in addition to fish (mixed diet) were found in eight communities, two of which were made entirely of mixed-diet individuals (figure 3). Mean (non-zero) association in each community varied from 0.18 to 0.71 (mean ± s.d.: 0.34 ± 0.12). Louvain's algorithm further identified 0 to 5 subclusters in each community (mean ± s.d.: 2.6 ± 1.4) (electronic supplementary material, table S5).

**Figure 4.**
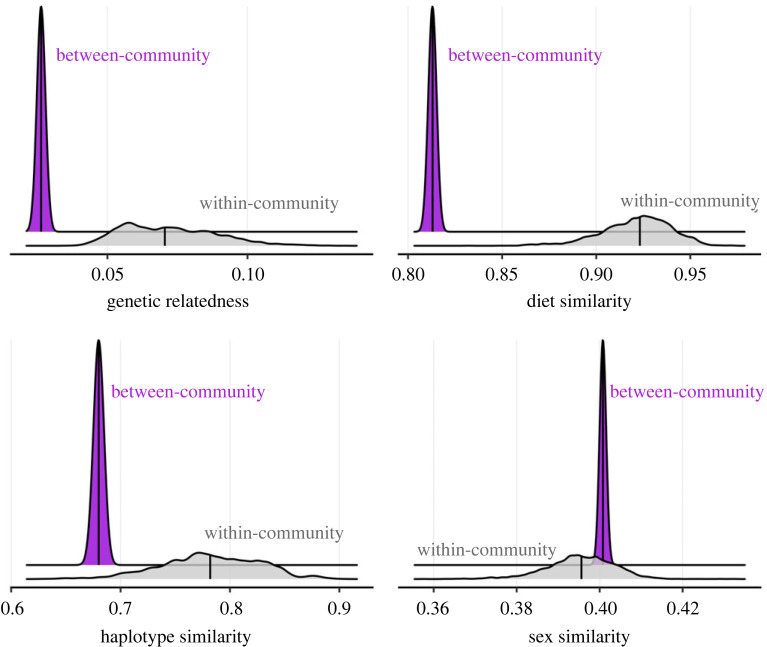
Posterior distributions of mean pairwise genetic relatedness, and dietary/sex/haplotype similarities within, relative to between, communities.

Average biparental genetic relatedness increased from (mean ± s.d.) 0.030 ± 0.051 at the network level, to 0.075 ± 0.069 (*n* = 9) in communities, to 0.135 ± 0.084 (*n* = 8) in subclusters (electronic supplementary material, table S6). From our posterior distribution of community assignments, mean pairwise relatedness was also higher within (0.072 ± 0.017) than between (0.026 ± 0.0002) communities (100% differences greater than 0). In addition, killer whales within communities showed higher similarity in haplotype identity (within: 0.782 ± 0.045 versus between: 0.680 ± 0.002, 98.4% of differences greater than 0) and diet (within: 0.922 ± 0.018 versus between: 0.813 ± 0.001, 100% differences greater than 0) than between communities, whilst no assortment by sex was identified (within: 0.396 ± 0.008 versus between: 0.401 ± 0.0003, 25.5% differences greater than 0) (figure 4).

**Figure 5.**
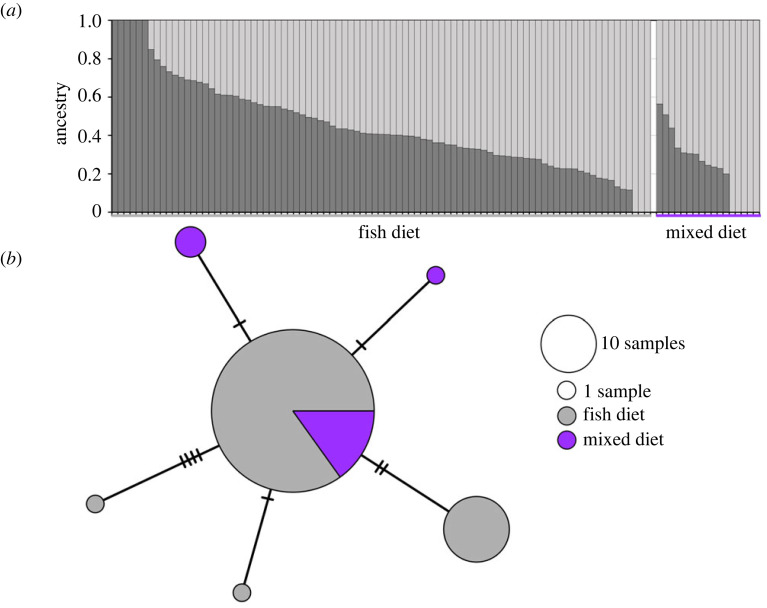
(*a*) Results from the NGSadmix analysis that modelled *k* = 2 populations. Each column represents a (unique) individual, and the shading (light versus dark grey) corresponds to individuals' proportions of ancestry attributable to each of the two putative populations. Killer whales with a fish diet are found to the left of the plot and mixed-diet individuals to the right. (*b*) Network of killer whale haplotypes generated from the mitogenome (16 390 bp). Each node corresponds to one haplotype, with size being indicative of the frequency of the haplotype and colour indicative of the diet group of the individuals they were sampled from. The number of lines between two nodes corresponds to the number of mutations between two haplotypes. Both analyses were based on all killer whales for which high depth of coverage data were available (Dataset 3: *n* = 106).

#### (iv) Number of populations

The NGSadmix analysis did not detect any genetic structure within the dataset, as the best supported model identified one unique population (*k* = 1) (figure 5*a*). A total of six haplotypes were identified among the 104 mtDNA sequences of unique individuals successfully analysed (figure 5*b*). Most individuals (*n* = 84) shared the same haplotype, including 13 of the 17 mixed-diet individuals. Another five haplotypes, with only one to four mutations difference from the main haplotype, were identified; two of these were found exclusively in mixed-diet killer whales.

## 4. Discussion

Our record of social relationships among individuals incorporating kinship and diet data, across multiple years and varied ecological contexts, generated an unprecedented approximation of the social landscape for an ecologically complex killer whale population. Our results revealed non-random patterns of association that correlated with kinship and diet. However, we found no evidence of social or genetic differentiation between individuals adopting different diets. Instead, we found a highly connected, yet fluid network of behaviourally diverse individuals, with kin-based associations at the core of basic social units.

Kinship was identified as the building block of killer whale sociality in Norway, aligning with previous findings of social groups organizing around matrilineal lineages in this species [9,56,57]. Maternal kinship explained the strongest associations, regardless of dietary group identity, especially between males and females. Age data (E. Jourdain 2008–2021, unpublished data) supported a mother–son relationship for these pairs, aligning with previous reports of the mother–son bond lasting way into adulthood both in resident [9,58] and in Bigg's killer whales [59]. Kin associations provide individuals with inclusive fitness benefits through group hunting [60], food sharing [61], cooperative care and altruism [62], and access to accrued ecological knowledge [10,11]. In our network, however, social bonds rapidly became variable with decreasing genetic relatedness as a result of common non-kin-based associations. Further work on dispersal patterns (e.g. similar to [59]), perhaps focusing on most documented groups to maximize statistical power, would help addressing the nature of the weaker and/or non-kin based ties observed in our network.

Dietary similarity was found to also be a correlate of association. However, because feeding behaviours are culturally transmitted within kin-based killer whale groups (e.g. [63]), the presence of clusters of highly associated, genetically related whales sharing the same diet in our data was likely a consequence, rather than a driver, of association—i.e. a result of cultural diffusion in the network (see [18]). Nevertheless, we cannot rule out some level of true social assortment by diet, as preferentially associating with others that adopt similar feeding techniques can provide benefits for cooperative behaviours [5].

Though most pairwise associations in our network were weak, we identified several communities of highly associated individuals. Within communities, strength of association among individuals adopting similar or different diets varied widely, with subclusters defining most cohesive social units found in near constant association. Such variation in the strength of association within communities suggested the existence of both highly persistent and temporarily preferred associations. Nevertheless, kinship correlated well with network subdivision. Higher genetic relatedness measured within, relative to between, communities suggested kinship also influenced casual but preferred associations between genetically related core social units (subclusters). This was further supported by mean genetic relatedness decreasing from subcluster to community to population. Taken together, our findings of flexible associations within and between ecologically diverse social units were more comparable with the fluid fission–fusion social structure not completely assorted by diet, documented for killer whales of Iceland [64,65] and Marion Island [66,67], rather than the strict sociality within foraging specialists of the North Pacific [8,9,57].

Fluidity in social affiliations could be a response to dynamic ecological/foraging conditions, i.e. a result of changing prey type/distribution/behaviour over time affecting optimal group size [56,64,66,68–71]. For example, in our dataset some individuals known to seasonally switch between fish prey resources are found in smaller groups when individually foraging on scattered lumpfish in spring than during coordinated feeding on abundant herring in winter [21,22]. Adjusting group size while maintaining familial ties may maximize energetic gain while maintaining the fitness benefits of kin-based social units [10,11,64,68]. In shared habitats where prey types co-occur, prey-switching killer whale groups can temporarily interact with other groups to reach foraging benefits without experiencing reduced fitness, further promoting flexible associations, including among non-kin.

Mixed-diet individuals, found in 8 of the 27 communities, were widely distributed across the network. The existence of associations between these and the whales adopting an exclusive fish diet highlighted the lack of social segregation based upon dietary preference, as could have been promoted by cultural barriers [5]. Consequently, analyses of population structure based on biparentally and maternally inherited genetic markers identified no structuring based on dietary variation in our dataset. This was consistent with the low genetic differentiation for killer whales with different but overlapping diet/ranging patterns from Iceland [72], but in sharp contrast with genetic differentiation of North Pacific ecotypes [12–14,32,37,40,57,73].

**Figure 6.**
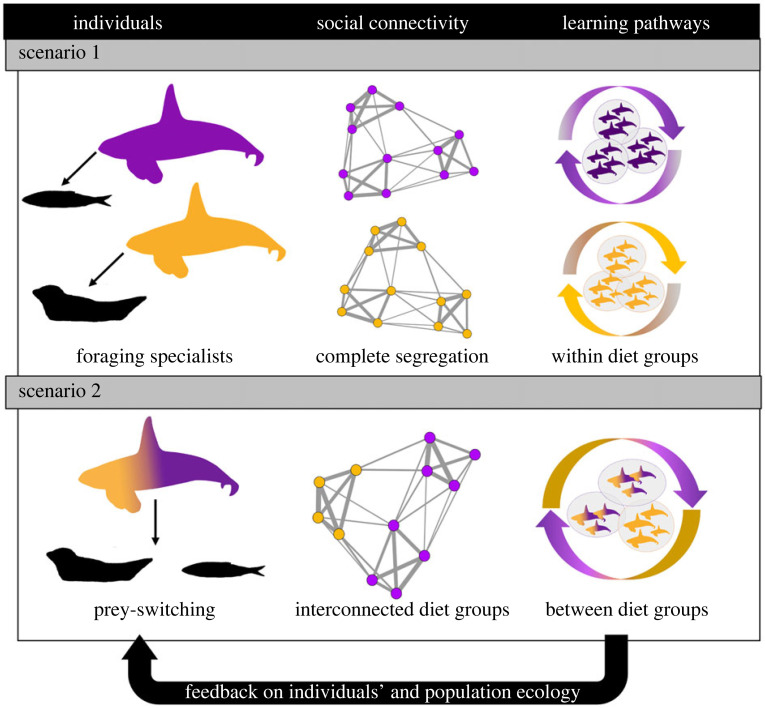
Conceptual visualization of the ecological (inter-individual dietary variation) and social mechanisms (connectivity and social learning) that may interact to influence individual behaviour and population-level ecology into strict foraging specialists (scenario 1, e.g. North Pacific) or prey-switching (scenario 2, e.g. Norway) in killer whale societies.

Our network of killer whales in Norway had comparable modularity (*Q* = 0.68) to that reported within foraging specialists of the North Pacific (southern residents: from 0.63 to 0.84 in [74]; Bigg's: 0.523 in [75]), and generalist populations off Marion Island (*Q* = 0.66, [67]) and Iceland (*Q* = 0.68, [65]). For all five populations, such modular network structure allows cultural perpetuation of behaviours within most cohesive social units, while weak but existing social ties between units provide opportunities for social learning and behaviours to spread (e.g. [76]). However, the absence of social contact between *resident* and *Bigg's* killer whales restricts social learning to occurring within ecotype/population only. In contrast, within a densely connected network of behaviourally diverse individuals and groups, killer whales off Iceland, Marion Island and Norway may be exposed to a broader behavioural repertoire that, through cultural transmission, contributes to a wider diet breadth at the population level. We illustrate this concept in figure 6.

Our study of observed associations could be missing or underestimating important ties of the true social network. However, our dataset had enough resolution to reveal social connections between ecologically diverse individuals/groups, challenging the long-standing conventional view of ecology driving killer whale sociality. Taken together with other killer whale studies (e.g. [65,67]), our findings point to a role of the social landscape in shaping populations' ecology. Specifically, social connectivity, ecological conditions and the emergence of behavioural innovations may all contribute to the balance between individual and population dietary variation or specialization. As long-term multi-disciplinary datasets become available for new populations, killer whale societies are a promising model to explore the relationship between network properties, behaviour dynamics and populations’ ecology.

## Data Availability

Association data are available from Figshare Data Repository [77]. Sequencing data are accessible at the NCBI BioProject PRJNA956724: MULTIWHALE (https://www.ncbi.nlm.nih.gov/bioproject/PRJNA956724/). For access to the database of killer whale sighting histories, contact the Norwegian Orca Survey.
